# Multiculturalism is good for flies, too

**DOI:** 10.1371/journal.pgen.1007480

**Published:** 2018-07-19

**Authors:** J. Robert Manak

**Affiliations:** 1 Department of Biology, University of Iowa, Iowa City, Iowa, United States of America; 2 Department of Pediatrics, University of Iowa, Iowa City, Iowa, United States of America; University of Arizona, UNITED STATES

*Drosophila melanogaster* has been used for several decades as a proxy to study human disease; in particular, flies are ideal for modeling neurological disorders (e.g., epilepsy, neurodegeneration, ataxia), as flies utilize the same neural circuitry components [1]. For example, both excitatory and inhibitory neurons form elaborate neural circuits that function through conserved ion channels, neurotransmitters, receptors, etc. Perhaps more remarkably, flies have also been used to study genes associated with intellectual disability and autism, using genetics coupled with learning assays to functionally validate genes implicated in these neuropsychiatric disorders [2]. Collectively, these studies have shown that many of the genes involved in vertebrate learning and memory are playing similar roles in flies. But what about aspects of cognitive function as it relates to social communication, processes that are mostly attributed to vertebrates with more complex nervous systems and insects such as ants and honey bees that are highly social [3,4]? Fruit flies certainly have the ability to learn (and remember what they’ve learned), but can they teach vital information to other members of their own species? What about members of different *Drosophila* species? How might information be conveyed in species that are separated by significant evolutionary distances? If communication exists, what parts of the brain are required, what gene functions are involved, and to what extent does neural plasticity play a role? In the current issue of *PLOS Genetics*, Kacsoh and colleagues [5] now demonstrate that flies not only can convey a threat from a predatory wasp to members of their own species but can convey that threat to members of several diverged species, provided that the flies are cohabitated and can learn each other’s dialect (even if multiple species are included in the cohabitation period). These data reveal a level of behavioral complexity in flies that has not been recognized until now.

## Flies use visual cues to convey a threat, resulting in a physiological response

Confirming results from their previous study, Kacsoh and colleagues first show that female flies exposed to predator wasps (which lay eggs into and kill the offspring of the female flies) physiologically trigger apoptosis of oocytes in the germline of adults, leading to reduced egg laying (oviposition) and a robust reduction in the wasp threat to the fly population. Interestingly, female flies that have experienced this threat can teach naïve “student” female flies (who have never been exposed to wasps) that there is a threat, resulting in the student flies then triggering apoptosis of their own germ cells. This threat is conveyed using visual cues from the teacher flies, more specifically, wing movements. These results are remarkable for three reasons. One, this is the first example in flies of visual cues promoting a robust, physiological response to a threat. Two, laboratory cultures that have not experienced a wasp threat for hundreds of generations can still mount a vigorous response to the threat. Three, the apoptosis occurs at the midoogenesis checkpoint rather than during both midoogenesis and early oogenesis (the points where apoptosis has been correlated with nutritional deprivation stress) [6], suggesting that there is a specificity to the information that is conveyed and that wing movement–encoded information for a wasp stressor appears to activate a different pathway than for nutritional stress. Although not included in the study, it would be interesting to assess whether nonparasitoid wasps (or other potentially threatening insects dissimilar to fruit flies) would elicit an apoptotic response in the fruit fly teachers. If not, this would strengthen the conclusions and suggest that the kind of information conveyed from teacher to student is not simply a “high stress” signal but a signal that can convey the type of stress in order to mount a specific physiological response.

## Related fly species communicate using a conserved “fly language”

How evolutionarily conserved is this response from species to species? Studying 15 different species that widely differ in their evolutionary relatedness, Kacsoh and colleagues found strong conservation of the response for all species tested, resulting in apoptosis and depression of egg laying after exposure to a wasp. Each species could also teach the threat to other female members of that species, once again resulting in depressed egg laying. Moreover, a wasp-exposed female from one species could teach students from a related species, although the ability to teach was somewhat diminished. In contrast, significantly diverged species could not teach the threat to one another. Collectively, these data suggest the existence of a “fly language” that has been conserved across evolutionary time, although communication depends on a certain level of evolutionary relatedness.

## More distantly related fly species learn each other’s language dialect in order to communicate

**Fig 1:**
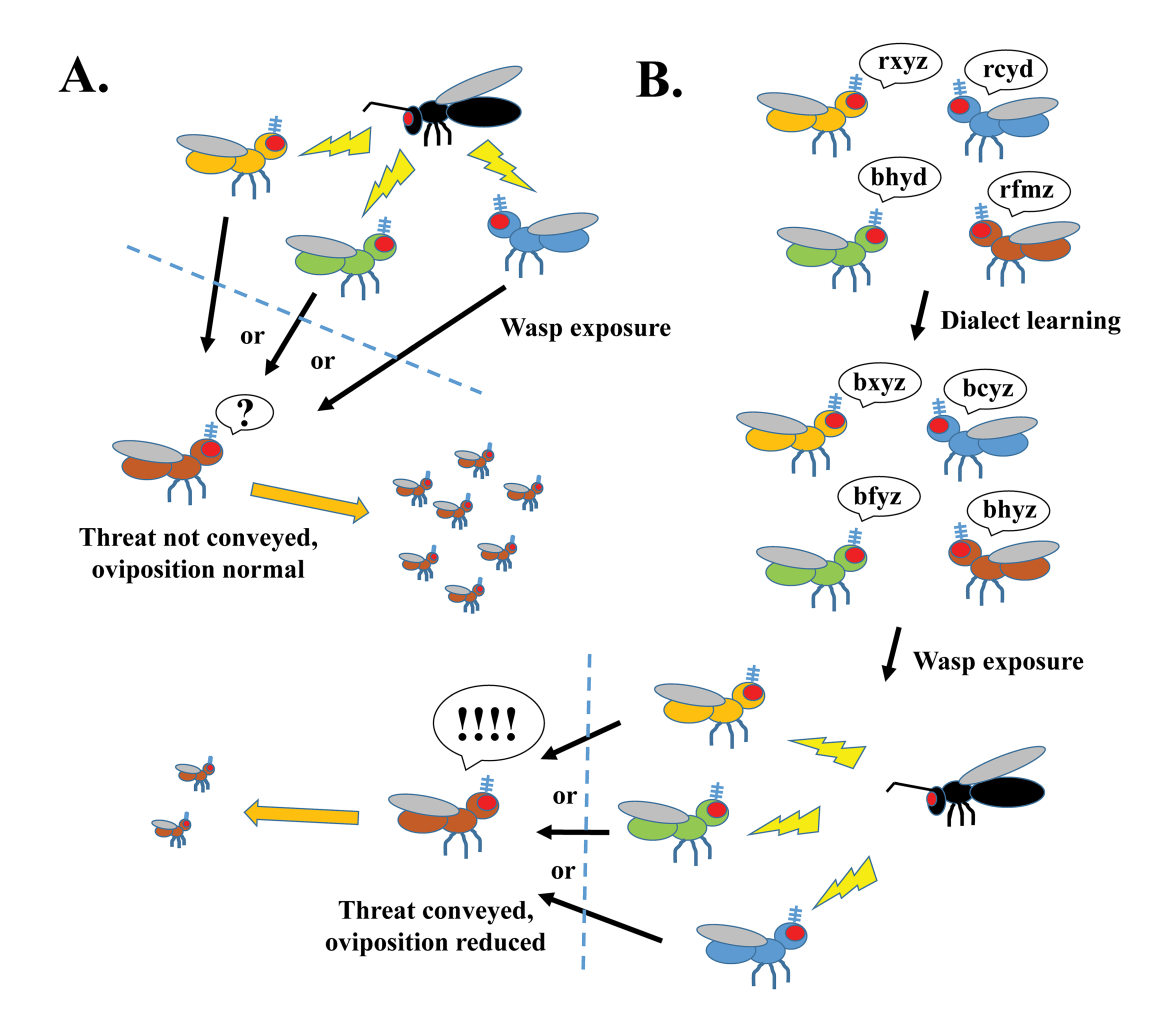
Generalized schematic for dialect learning. (A) Female flies cannot efficiently communicate a wasp threat if “teacher” and “student” are from different diverged species. (B) Cohabitation of diverged species allows for each species to learn the language “dialects” of the others; once dialects are learned, a female teacher now can communicate a wasp threat to a female student from a diverged species. Different diverged *Drosophila* species are highlighted in different colors (orange, green, blue, brown). Wasp is highlighted in black.

Previous work in bees has shown that social learning helps bees teach their hive mates how to obtain a reward or achieve a task [7,8]. However, bees are “social” and known to work in groups, unlike fruit flies that tend to work in isolation. Could the cohabitation of two different diverged fly species actually improve (or even enable) these species to talk to one another, allowing one species to convey the wasp threat to the other? Kacsoh and colleagues now show this to be the case and refer to this process as “dialect” learning. Remarkably, cohabitation of several diverged species together results in student flies learning and remembering each unique dialect, suggesting that flies can simultaneously learn from multiple species (Fig 1). Trained flies can then be taught there is a wasp threat by teachers of any of the other diverged species (provided that the evolutionary distance between teacher and student is not too great). What is most intriguing about this cohabitation training period is that none of these flies have previous experience with the predator, so whatever exchange of information is occurring during a dialect training period, it appears to be sensitizing different species to more efficient communication of future and yet unknown events.

## Both visual and olfactory cues are required for dialect learning

Since students can learn from teachers using exclusively visual cues, is this also true for dialect learning? Although visual cues were shown to be required (specifically, wing movements), so were olfactory cues, making dialect learning different from teacher–student learning. Interestingly, if one species is blind, the sighted species cannot learn from it, suggesting that communication is bidirectional in that perception of other species must be necessary to elicit behaviors or signals required for dialect training. In addition to visual and olfactory components, both sexes are also required to be present for dialect training another species, making this process all the more complex.

## Neuroplasticity is critical for dialect learning

To explore whether the fly learning and memory center of the brain, the mushroom body (MB), is required for dialect training, Kacsoh and colleagues inhibit MB function only during the training cohabitation period, and although the MB-inhibited flies cannot learn the dialect from the other species members, those with normal MB function can learn from the MB-inhibited flies. Knockdown of long-term memory proteins produces similar results, with learning only possible in one direction (in the species for which long-term memory proteins are not affected). The requirement of long-term memory functions and an intact fly learning and memory center, coupled with the results described above, suggest that in addition to hardwired components, neuroplasticity is critical for dialect learning. These observations also suggest that continual and real-time interactions between different species are sufficient for eliciting signals required for a productive training cohabitation period in a manner independent of MB learning and memory functions and that memory is necessary only after the training period is complete.

This study reveals that fruit flies may not only have a language with which to communicate but also have natural variants akin to dialects associated with different closely related species and that those dialects can be learned, thereby facilitating communication of critical information that can influence the survival of multiple species. These interesting new observations were enabled by the simple experiment of mixing different species together, something that in nature occurs all the time but is rarely done in the laboratory. Maybe the “lowly” fruit fly, when studied closer to its natural environment, can teach us all something about the benefits of multiculturalism, namely, that by communicating with one another and working together, we can create a more productive and safe society.
